# The porin AaxA protein model from Chlamydia pneumonia

**DOI:** 10.6026/97320630016786

**Published:** 2020-10-31

**Authors:** Jayaraman Selvaraj, Srinivasan Perumal, Josephine Rex, Surapaneni Krishna Mohan, Sumetha Suga Deiva Suga, Umapathy Vidhya Rekh, Veeraraghavan Vishnupriya, Periyasamy Vijayalakshmi, Rajagopal Ponnulakshmi

**Affiliations:** 1 Department of Biochemistry, Saveetha Dental College and Hospitals, Saveetha Institute of Medical and Technical Sciences, Saveetha University, Chennai - 600 077, India; 2 Department of Biochemistry, School of Life Sciences, Vels Institute of Science Technology and Advanced Studies (VISTAS), Pallavaram, Chennai- 600 117, India; 3 Department of Biochemistry, Panimalar Medical College Hospital & Research Institute, Varadharajapuram, Poonamallee, Chennai - 600 123, India; 4 Department of Microbiology, Panimalar Medical College Hospital & Research Institute, Varadharajapuram, Poonamallee, Chennai - 600 123, India; 5 PG & Research Department of Biotechnology & Bioinformatics, Holy Cross College (Autonomous), Trichy- 620002, Tamil Nadu, India; 6 Central Research Laboratory, Meenakshi Academy of Higher Education and Research, Chennai-600 078, India; 7; 8

**Keywords:** Chlamydophila pneumonia, AaxA, homology modelling

## Abstract

Chlamydophila pneumoniae is an intracellular pathogen accountable for various acute respiratory infections. C. pneumoniae has a gene cluster which encodes a putative outer membrane porin (aaxA), arginine decarboxylase (CPn1032 or aaxB) and a putative cytoplasmic membrane transporter (CPn1031 or aaxC). Therefore, it is of interest to document a molecular protein model of porin AaxA from Chlamydia pneumonia to gain structure to functional insight on the protein.

## Background

Chlamydophila pneumonia is a species of Chlamydophila. It is a microscopic, gram negative, intracellular bacterium that infects humans. It is the main cause of pneumonia. C. pneumoniae is transmitted directly from person to person through the respiratory system [1-2]. The incubation time is several weeks longer than that for several other respiratory pathogens [3]. C. pneumoniae is capable of developing an intracellular niche where it facilitates the survival or death of the host cell, modulates the hormonal signalling pathway of the host cell, and bypasses the defensive mechanisms of the host cell. C. pneumoniae causes a persistent infection due to the inability of the host to remove the pathogen [4-6]. Porin AaxA protein enhances the absorption of L-arginine as part of the AaxABC system. Therefore, it is of interest to document a molecular Porin AaxA protein model from Chlamydia pneumonia to gain functional insight.

## Materials and Methods:

### Template sequence Alignment:

The porin AaxA protein sequence was obtained from the Uni-protKB/Swissprot database (Q9Z6M6) [7]. The Brook Heaven Protein Data Bank (PDB) was used to find the correct template (PDB ID: 2P3N) for modelling the porin AaxA protein with default parameters. The alignment between the target and the template (identity score of 37%) was done using the omega cluster [8].

### Molecular Modelling of porin AaxA protein:

The homology of the porin AaxA protein modelling was conducted using the template structure with PDB ID: 2P3N using the modeller9v9.19 software [9]. PYMOL was used to visualize the modelled structure [10]. All modelled structures were graded on the basis of the internal score function (DOPE score). Models with the lowest internal score were chosen as the final model for accuracy assessment [11,12].

### Validation of the model:

The model was evaluated using energy and stereo chemical geometry [13]. The stereo chemical consistency of the modeled protein was checked using the Phi / Psi distributions in the Ramachandran plot generated with PROCHECK in SAVS (Structure Analysis Verification Server) [14].

## Results and Discussion:

**Figure 1:**
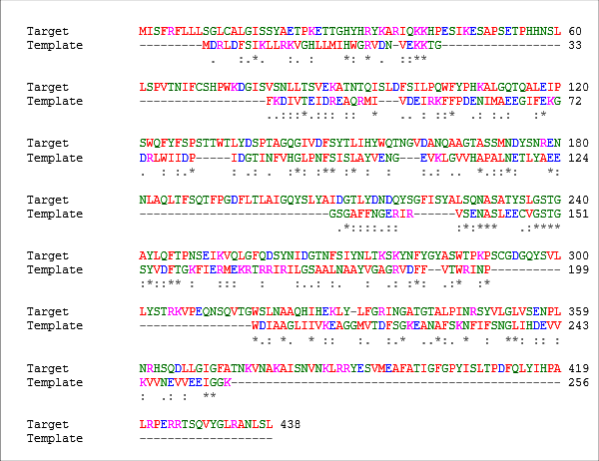
Alignment of porin AaxA with template (PDB: ID2P3N) protein sequence using clustal omega. Asterisks indicates identical amino acids, dots indicate similar amino acids.

**Figure 2:**
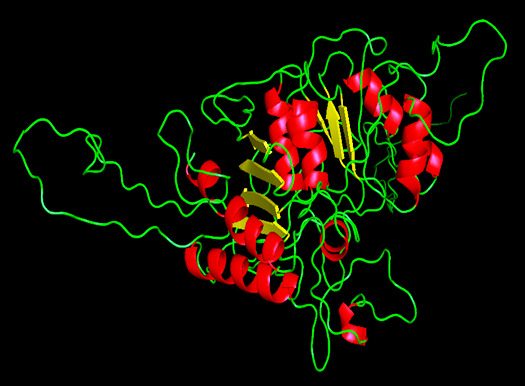
The best modeled structure of porin AaxA protein obtained using Modeller 9v9.19. Red colour indicate alpha helices, yellow colour indicate the beta sheets and green colour indicate the loops

**Figure 3:**
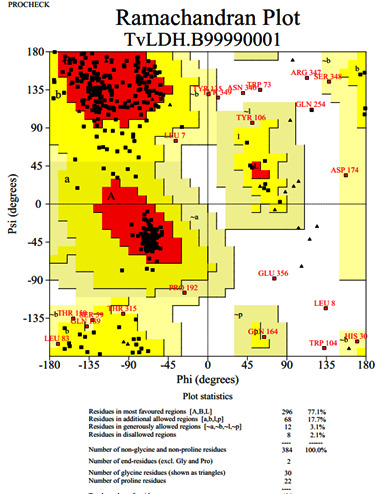
Ramachandran plot for the porin AaxA protein obtained using PROCHECK

The target protein sequences (porin AaxA) and the template (PDB ID: 2P3N) were matched and the alignment result was shown in Figure 1. The asterisk indicated the identity of the amino acids found in the two protein sequences. MODELLER9.19 was used to generate a model for the target protein porin AaxA (Figure 2). We selected TvLTH.B99990001.pdb porin AaxA protein model using the minimum dope score rating. SAVS (Structure Alignment Verification Server) was used to verify the modelled structures. The measurements of the Ramachandran plot are computed using the PROCHECK software (Figure 3). The preliminary predicted model data is available with the authors for further analysis to gain functional insights on the protein.

## Conclusion

We report a molecular protein model data of porin AaxA from Chlamydia pneumonia to gain structure to functional insight.
